# The Role of the Food Banks in Saving Freshwater Resources through Reducing Food Waste: The Case of the Food Bank of Navarra, Spain

**DOI:** 10.3390/foods11020163

**Published:** 2022-01-09

**Authors:** Josemi G. Penalver, Maite M. Aldaya

**Affiliations:** 1 Arrosadia Campus, Public University of Navarra (UPNA), 31006 Pamplona, Spain; josemiguel.gonzalez@unavarra.es; 2 Arrosadia Campus, Institute for Sustainability & Food Chain Innovation (IS-FOOD), Public University of Navarra (UPNA), Jerónimo de Ayanz Building, 31006 Pamplona, Spain

**Keywords:** water footprint, food waste, sustainability, food bank, Spain

## Abstract

In the year 2011, the FAO estimated that food loss and waste reached one third of the total food produced worldwide. Since then, numerous studies have been published characterizing this problem and reflecting on its repercussions, not only social, but also environmental. Food wastage triggers unnecessary greenhouse gas emissions, deforestation or loss of biodiversity. This study aims to quantify the water-related benefits associated with food loss and waste reduction by studying the Food Bank of Navarra (FBN). For this purpose, the water footprint assessment manual has been followed. First, the water footprint of the activities of the FBN has been analysed for the year 2018 (scenario with the FBN). A comparative analysis has been carried out between the scenario with the FBN and a theoretical scenario without the action of the FBN. This has allowed us to highlight the benefits associated with the activity of this entity. The FBN not only avoided the waste of 2.7 thousand tons of food suitable for consumption in 2018, but also avoided the unnecessary use of more than 3.2 million m^3^ of freshwater. As a result of the present investigation, it can be stated that promoting food banks, which avoid food waste, would be an effective way to contribute to the protection and conservation of water resources.

## 1. Introduction

In recent years, there has been a growing concern about the problem of food loss and waste in the world. According the Food and Agriculture Organisation of the United Nations (FAO), globally, every year, one third of the food produced for human consumption is lost or wasted, which is around 1.3 billion tonnes/year [1]. Both developed and developing countries are responsible for this phenomenon. This situation represents a crucial target for improvement. Institutions, such as the European Union, have included recommendations and new strategies to prevent food waste in the European Green Deal and its landmark Farm to Fork Strategy [2]. Likewise, at a legislative level, in 2021, the Spanish government approved the first draft bill aimed to fight food waste. This law states that all the actors in the food chain must have a prevention plan to avoid food waste. It also sets a hierarchy of mandatory priorities, the first of which is the use of food for human consumption through donations to non-profit companies or food banks [3]. The reduction of food waste along the value chain would help to increase global food security and mitigate the environmental impacts generated by the agri-food sector [4].

Food banks come into the picture as a solution to this problem, as their activity is directly related to preventing the waste of food that is fit for consumption throughout the entire production chain. These entities are non-profit organizations that collect and distribute food to hunger-relief charities or directly to people struggling with hunger. Food banks act as storage and distribution depots for food that is not commercialized by the food producer or distributor, is poorly packaged or is close to the expiration date but fit for human consumption, or came from donation campaigns. The work is usually carried out by volunteers, but sometimes these organizations are funded by public and/or private institutions. The world’s first food bank was established in Phoenix, AZ, USA, in 1967. Since the 1980s, food banks have spread around the world. There are over 30 countries with active food bank groups under the umbrella of The Global FoodBanking Network [5]. Food banks are increasingly necessary as, in the last years, under the COVID-19 context, food insecurity has increased to its highest levels in decades [6].

In particular, the Food Bank of Navarra received and distributed around 2700 tonnes of food in 2018. Its mission is, in addition to improving the food security of people in a situation of social exclusion and/or poverty, to involve society and companies in the rationalization of food use and consumption, as a potential impact factor for environmental sustainability.

There are several studies that analyse the environmental impacts associated with food waste along the entire food supply chain. They include impacts related to global warming, water, photochemical ozone formation, eutrophication, human toxicity, fossil resource depletion, acidification, particulate matter and eco-toxicity [7,8,9,10,11,12]. The lower the food waste, the lower the environmental impact. Particularly, food waste has a significant impact on freshwater use, as agriculture is responsible for over 90 percent of global water consumption [12,13]. Several studies have quantified the impacts of food waste on water resources in different contexts and terms [9,10,11,12]. There is only one study published by Reynolds et al. [14] that has quantified the positive effects of food rescue at the macro level, at the national level in Australia, applying input–output analysis. According to this study, every US dollar spent on food rescue and its redirection, saves 6.6 m^3^ of freshwater. Just a few studies, such as those carried out by Aldaya et al. [15], Guilhem [16] or López et al. [17], highlight the environmental benefits of food bank activities, focusing on the minimization of greenhouse gas emissions and their positive influence on climate change. To our knowledge, there are no publications quantifying the impact of food banks’ activities on freshwater consumption and pollution.

Spain has been ranked as the country with the third-highest water exploitation index (WEI) score in the European Union, surpassed only by Greece and Cyprus. In turn, it is estimated that in recent years (1990–2017), the amount of renewable freshwater per inhabitant has been reduced by up to 65% [18]. These numbers show the urgency of implementing a good water resource management policy in this country. Numerous authors have offered similar perspectives on the situation of water resources in this country and their management, as well as possible solutions to minimize the pressure exerted on this natural resource, proposing the reuse of wastewater [19], investment in the development of more efficient desalination plants [20] or changes to the irrigation systems and crop selection [21,22,23]. Of all the sectors and industries with a significant impact, agriculture is the main sector responsible for the use of this resource, consuming around 85% of the water resources located in Spain [24].

The present paper aims to analyse, for the first time, the impact of the food banks, by reducing food waste, on freshwater consumption and pollution. With this purpose, first, this work assesses the water footprint generated by the activities of the Food Bank of Navarra (scenario with the FBN). Second, it analyses the water use in a scenario without the FBN (scenario without the FBN). Hereafter, a comparative analysis of the two scenarios, with and without the FBN, is conducted, confirming the water-related environmental benefits associated with the activity of these kind of foundations. Even if certain limitations were found in terms of data availability, the objective data provided by the study clearly show the need for further reducing food waste by using food banks and other actors along the food chain.

## 2. Materials and Methods

### 2.1. Research Strategy and Water Footprint Assessment

The water footprint of the Food Bank of Navarra was compared with the water use in a hypothetical situation without the action of the Food Bank in the year 2018. To this end, first, the water footprint of the Food Bank of Navarra was assessed (scenario with the FBN). Second, the water use in a theoretical scenario without the existence of the FBN was estimated (scenario without the FBN). Finally, a comparative analysis of both scenarios was performed.

The scenarios designed for this study were agreed upon with the FBN and are similar to the ones used in the assessment of the carbon footprint of food banks [15,16,17]. In order to analyse the water use in both scenarios, the water footprint methodology of the water footprint assessment manual [20] was followed. An ad hoc Excel-based water footprint calculator was developed for the realization of this study.

The water footprint (WF) is a consumption-based indicator of water use, which quantifies and characterizes the water used by a consumer or producer. A company-focused WF, which can also be referred to as a corporate or organizational water footprint, is defined as the total volume of freshwater used directly and indirectly for the development of the company activities. This water footprint of an organization is made up of two components: the direct or operational water footprint (for production/manufacturing or support activities) and the indirect or supply chain water footprint (water used in the producer’s supply chain). Additionally, it has three colours: green water footprint (the consumption of rainwater that does not become run-off), blue water footprint (the consumption of ground-surface water) and grey water footprint (the volume of freshwater required to assimilate a pollution load) [25].

First, to evaluate the water footprint of the activities of the FBN, and to determine the possible impacts of this organization on water resources, a direct communication with the FBN was maintained. The activities under evaluation in this part of the study include: freshwater directly used in the FBN’s daily activities, freshwater consumption related to the production of the energy consumed, freshwater invested in the production of the FBN goods and finally, the water expenditure related to the transport of this food to the FBN’s facilities and distribution centres, and the transportation of staff and volunteers.

Second, in the theoretical scenario without the existence of the FBN, two main sources of impact have been taken into account: (1) the water consumption and pollution due to the treatment of the disposed food, which would not have been redistributed by the FBN, and (2) the water use for the production of new food products to replace the wasted ones.

**Figure 1:**
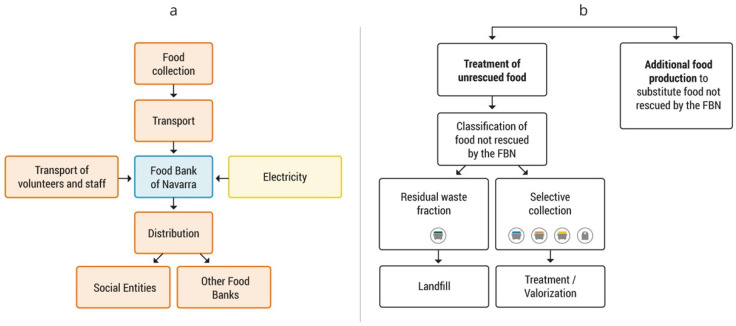
Activities considered in the two scenarios: (**a**) scenario with the Food Bank of Navarra (FBN); (**b**) scenario without the FBN. In the scenario with the FBN, the food collection points included: manufacturers and distributors, The Fund for European Aid to the Most Deprived (FEAD programme), large-scale collections, Pamplona Region Commonwealth, Fruit and Vegetable Producers Organisation, other food banks and local collections.

Finally, the comparative analysis of both scenarios determined whether the actions of the food bank have negative or positive effects on water resources. The mentioned analysis compares the scenario that actually occurred in 2018, with the FBN in full operation, and a theoretical scenario without the existence of the FBN. Although these scenarios are opposed, it should be noted that some activities with an impact on water resources may occur in both scenarios, such as the use of water for waste management, generated after the consumer phase (impacts by the consumer [26]), or the transport from the food distribution entity to consumers’ houses. This type of activity would entail an expenditure on water resources, but as the same water use is considered in both scenarios, its determination has no value for the comparative analysis, which would not be affected. The activities considered in each scenario are shown in Figure 1.

### 2.2. Scenario Analysis: Data Collection and Data Analysis

#### 2.2.1. Scenario with the Food Bank of Navarra

**Figure 2:**
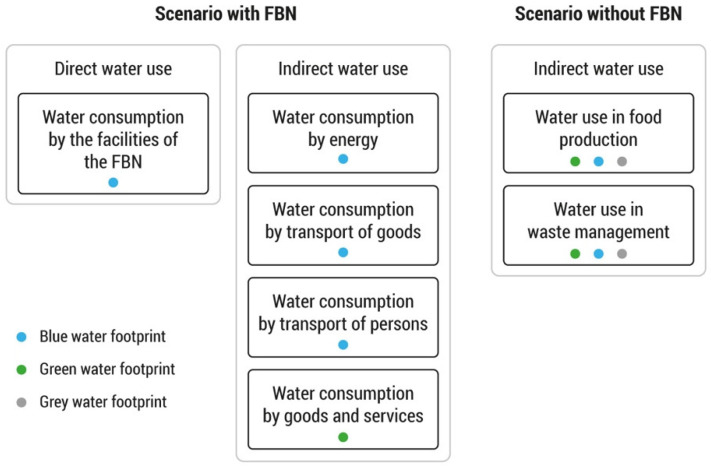
Direct and indirect water uses taken into consideration within the boundaries of the study in a scenario with the Food Bank of Navarra (FBN) and a scenario without the FBN.

The green, blue and grey water footprints of the Food Bank of Navarra were assessed for the year 2018 following the water footprint assessment manual [25]. The water footprint of the activities of the FBN consists of direct or operational water use and indirect or supply chain water use, as shown in Figure 2.

The direct water footprint refers to freshwater use in the FBN headquarters in Pamplona and Tudela, which includes the consumption of drinking water and water use in auxiliary activities, such as cleaning or sanitation. This was calculated according to the regional legislation of water use for domestic houses and small companies in Navarra. This legislation considers that all the water used by a small organization is to be subsequently treated and returned to the original watercourse or public water network [27].

The indirect water use includes the transportation of food donated to both FBN headquarters, its subsequent distribution to social entities or other food banks, the transportation of the staff necessary to carry out the activities and the use of water for the production of the energy consumed and the goods used (Figure 2).

The indirect water footprint of energy is related to the production of natural gas, diesel and electricity provided by two companies: EMASP S. Coop and Iberdrola Clientes. The Appendix A Table A1 includes the sources for the conversion factors used for each energy type. All of them considered the consumption of blue water in the process of energy production. For example, the water evaporated from manmade reservoirs to produce hydroelectricity or the water consumed in the cooling cycles of nuclear, gas and coal-fired power plants [28].

Regarding transportation, the freshwater consumption associated with the production of fuel oil used by the FBN vans was calculated assuming the following. Data published by Don Hofstrand [29] were used to estimate an energy average of 0.0359 GJ per litre of diesel (assuming the lowest calorific value, which is the most common in Europe). The conversion factor of 0.032 m^3^ of blue water per GJ of diesel produced was taken from Berger et al. [30].

The data for the transportation of the staff, the transportation of the foodstuff from the seven collection points to the FBN, and their transfer from the FBN facilities to the distribution entities or other food banks, was expressed in kilometres travelled per vehicle. Only in some specific cases was the fuel used specified. The literature shows that 1 litre of petrol and diesel is equivalent to the production of 0.032 and 0.0359 GJ, respectively [29]. The consumption factors of the different types of vehicles and brands were obtained from international databases in order to be able to convert the kilometres travelled into litres of fuel used, and then into m^3^ of blue water used [31,32]. The proportion of diesel and petrol used by the different vehicles was taken from Spain’s vehicle fleet in 2018 [33]. An average occupancy of 24 passengers is considered to divide consumption among public transport users, according to MITECO data (2020) [34]. It has been considered that 100% of the buses in Navarra used diesel, disregarding the 1.6% that were run in 2018 with something other than this fuel [33].

Finally, the water footprint related to the production of paper and cardboard in Spain, which were used as industrial packaging material for the transport and storage of food, were provided by Schyns et al. [35] (Appendix A Table A2). It comes entirely from the consumption of rainwater necessary for the growth of forests, according to the most common wood production systems in Spain [35].

The data on the consumption of goods and services were provided by the FBN in the form of litres of water consumed, kilograms of goods, kilometres of distance travelled by the different vehicles and kilowatts of energy consumption.

The water footprint of the building materials and vehicle construction was considered negligible, following Jefferies et al. [36].

#### 2.2.2. Scenario without the Food Bank of Navarra

In this theoretical scenario, the food redistributed by the FBN would have been disposed of, with the subsequent valorization treatment or with controlled discharges. On the other hand, additional food production would have been necessary to meet the needs of those users who benefited from the FBN during the year 2018 (Figure 1b).

In the case of waste management, the origin of the food came from three different places: 28.1% came from the Pamplona Region Commonwealth, 30.8% from other parts of Navarra and 41.1% from the rest of Spain. For each location, the most commonly used waste disposal or valorization systems and the most frequently encountered waste fractions were used.

In the case of the other parts of Navarra, the “Inventory of household and commercial waste” of the Government of Navarra for the year 2018 [37] was used to obtain the kind of waste treatment or disposal method employed. For the Pamplona Region Commonwealth characterization, the above-mentioned study was used in combination with the results of the study, “Characterization of household waste 2018” [38], also conducted by the Government of Navarra.

For the fraction coming from the rest of Spain, several autonomous communities were consulted in order to find out which waste management treatments were applied by the FBN providers in 2018. However, data were only obtained for the region of Catalonia and Navarra. Due to the absence of public data on waste management in most of the autonomous communities, an average of the data received from Catalonia and Navarra was assumed for the waste management systems in the rest of Spain.

Regarding water use in waste management, literature and some companies were consulted in order to obtain an estimation of the amount of freshwater required in the different waste management systems (Appendix A Table A3). The energy consumed in these processes has not been considered to be within the scope of this study.

On the other hand, the FBN redistributed a total of 2.7 thousand tonnes of food in 2018. In order to estimate the water that would have been invested in additional food production, the production of the same food that was redistributed has been assumed. Literature and international databases were consulted for the characterization of the production water footprint for each food category (Appendix A Table A4). The water footprint of food production includes the water directly added to the product, the rain and irrigation water consumed in the agricultural phase and the water polluted by nitrogen fertilizers). In those cases where the food was unclassified, a weighted average of the water footprint of all products has been used.

Wherever possible, reference data for Spain have been considered. For foodstuffs from livestock or basic agriculture that are not produced in Spain, global average values have been used. In the case of foods of complex formulation, such as broths, soups, gazpacho, jams and similar, the definitions of the Spanish food code [39] were followed in order to estimate the proportions. For all products of animal origin, water use has been estimated as a weighted average of water uses in extensive and intensive livestock production systems [40].

The freshwater uses related to food production have very different origins. It includes, for example, water embodied directly in the product (drink, juices, smoothies, etc.), used in the growth of agricultural products (grain, legumes, feed), used for sanitary operations, used for the production of goods, electricity and fuels needed in the production process, etc. [40,41]. More information about the water use related to the production of food products can be found in the literature cited in Table A4 of the Appendix A.

## 3. Results

### 3.1. Water Footprint of the Food Bank of Navarra

The operational and supply chain water footprints associated to the FBN’s activities are summarized in the Table 1:

The absence of operational freshwater consumption at the FBN’s headquarters has been determined in accordance with the protocols of the different regional regulations. These protocols mandate that all the water withdrawn in domestic houses and small companies in Navarra returns entirely to its respective watercourse or public water network.

**Figure 3:**
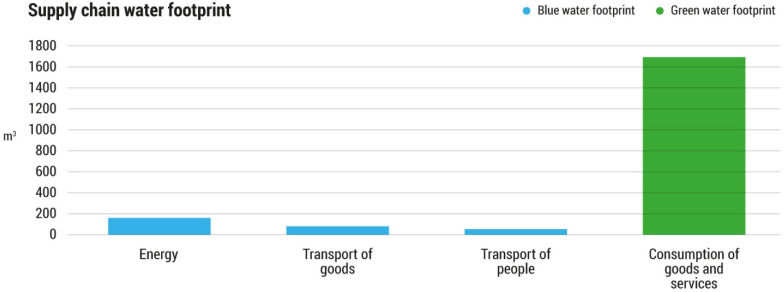
The supply chain green and blue water footprints (WF) associated with the Food Bank of Navarra’s activities, subdivided by source, in the year 2018 (m^3^).

With regard to the supply chain water footprint, the supply of cardboard and wood was the main source of water consumption, which amounted to 1685 m^3^ of green water. This was followed by the use of energy and means of transport, which involved the consumption of 241 m^3^ of blue water (see Figure 3).

### 3.2. Water Use in a Scenario without the Food Bank of Navarra

In this theoretical scenario, waste management would entail a total expenditure of 39,502 m^3^ of water (1% of the scenario without the FBN). Of this, approximately 11% is equivalent to the blue water consumed in paper recycling, and the remaining 89% is due to grey water, i.e., water polluted by leachates resulting from the dumping of food waste in landfills.

These values, however, underestimate the actual use of the freshwater needed to manage food waste, since data on the energy and materials used in the revalorization and controlled landfill processes were not available. Furthermore, the methodology of waste management systems can vary widely from one company to another, so water use data may vary if different companies are considered.

Food production in this scenario would be the main source of freshwater consumption and pollution, using 3.2 million m^3^ of water (99% of the scenario without FBN). The main cause of this consumption is rainwater (green water), which accounts for 73.3% of total water use in food production, and is mainly due to the production of agricultural products. Next, it is estimated that 17.1% comes from blue water consumption, mainly due to irrigation, and the remaining 9.6% refers to grey water related to the nitrogen used in the production of additional food.

**Figure 4:**
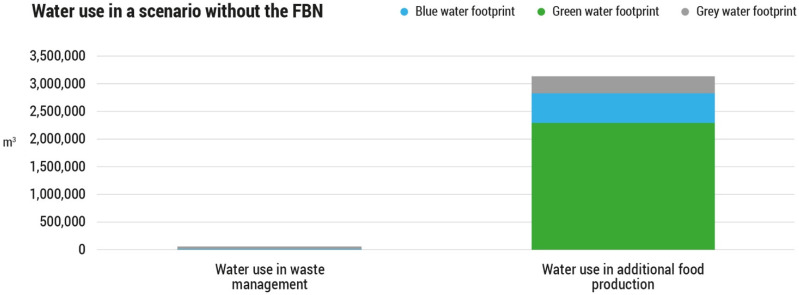
Green, blue and grey water use in the scenario without the Food Bank of Navarra in the year 2018 (m^3^).

Total freshwater use, in this scenario without the FBN, would amount to almost 3.3 million m^3^ of water, where green water accounts for 72.4%, blue water represents a 17.1% and the remaining 10.5% is grey water (Figure 4).

**Figure 5:**
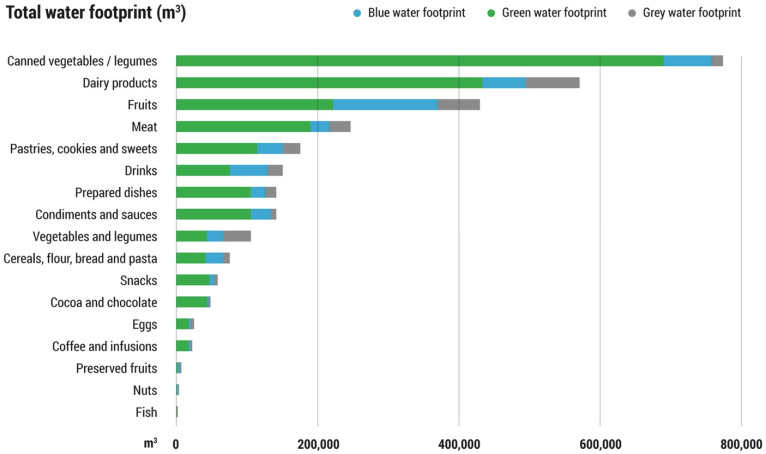
Green, blue and grey water footprints (WF) of every food category managed by the Food Bank of Navarra in the year 2018 (m^3^).

Figure 5 shows a representation of the water footprint of every food category considered, broken down by the type of footprint. Because of the lack of information about its composition, the water footprint of the categories “not catalogued” and “baby food” have not been taken into consideration.

The food categories that lead to the highest water use are those that were donated to the FBN in greater quantities. The three categories with the highest water use are canned vegetables/legumes, dairy products and fruits, which amount to 51.5% by weight of the food donated to the FBN in 2018. The exception is the fresh vegetables/legumes category, which is the second-most donated category, but whose components have very low water footprints.

### 3.3. Comparative Analysis of the Green, Blue and Grey Water Footprints “with” and “without” the Activities Food Bank of Navarra

The Food Bank of Navarra had an associated water use of 1926 m^3^/year, as shown in Table 1. In the absence of this organization, not only would 2.7 thousand tons of food have been wasted, but there would also have been a theoretical use of 3.2 million m^3^ of freshwater for the additional production of foodstuff and for waste management.

**Figure 6:**
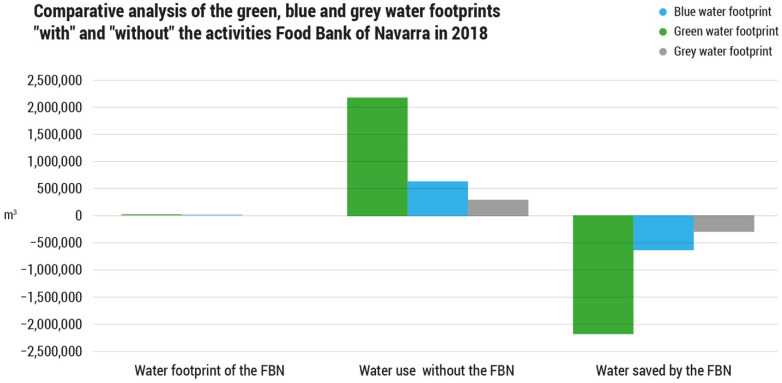
Comparative analysis of the green, blue and grey water use in the scenario “with” and “without” the activities Food Bank of Navarra in 2018 (m^3^).

Figure 6 shows the results of the water balance, broken down by type of footprint. Most of the freshwater “saved”, a total of 72.4%, corresponds to rainwater, followed by 17.1% of blue water, mainly due to irrigation, and the remaining 10.5% is grey water related to the nitrogen fertilizers used in the production of additional food. This indicates that the benefits associated with the maintenance of water resources is manifested in the different dimensions of its use.

## 4. Discussion

### 4.1. Implications of the Study

Many measures and actions have been put forward and implemented to reduce food waste. However, currently, the potential of the interventions to reduce food waste is only being assessed to a limited extent [42]. A clear understanding of the net benefits on the actual effectiveness of food waste measures is needed, not only from the socio-economic perspective, but also from an environmental viewpoint [42,43,44]. Our study quantifies the net benefits by assessing the activity of the Food Bank of Navarra in terms of reducing the water consumption and pollution. The comparative analysis of the scenario with and without the food bank has proven that the avoided water waste has been far greater than the waste provoked by the activities of the food bank. These results could be extrapolated to the rest of the food banks. This objective data obtained increases transparency and could create incentives for further reducing food waste by implementing further food banks and actors along the food chain.

On the other hand, in the literature there is a great variety in how measures are assessed [42]. The studies that quantify the impacts of food waste on water resources use different terms, including blue water consumption [10], water depletion and eutrophication [9], water scarcity [12,45] or green, blue and grey water footprint [11], among others. The present study uses the water footprint methodology, which represents a highly comparable and replicable tool to measure and quantify the environmental impacts associated with food loss and waste. Using robust indicators to measure performance is useful to monitor progress and make comparative analyses. This could enable practitioners and decision makers to compare food bank interventions, identify trade-offs and prioritize actions.

### 4.2. Limitations of the Study

The main limitation of the study has been data availability on the waste management systems in the scenario without the FBN. First of all, the absence of public data on waste management for the different Spanish communities has been a handicap for the realization of this study. We extrapolated the data of two autonomous communities, meaning that our data on waste management systems used in food wastage outside Navarra is not entirely accurate. As regards waste management within Navarra and the Pamplona Region Commonwealth, two organizations were consulted and taken as representative: HTN Biogas plant for the biomethanization, and the public Arazuri composting plant for the compost. It should be noted that the methodology of waste management systems can vary widely from one organization to another, so data on water use may vary depending on the organizations considered.

Furthermore, we were not able to find the energy consumption data for the different waste management systems, so the water expenditure associated with these has not been accounted for. In any case, the contribution of the energy factor would be a small percentage of the overall water footprint, as the Spanish electricity system energy mix [46] has just a small percentage coming from biofuels and hydropower, which account for 13% of the total energy produced. Moreover, according to the water footprint network manual [25], the energy factor does not have a relevant influence on the water footprint as long as biofuels or hydropower are not used as a source. In conclusion, the total water footprint values would be just slightly underestimated in our study.

Finally, there are many uncertainties related to both the usage of water for the treatment of the waste generated after the consumption of the rescued food in the scenario with the FBN and the treatment of the waste from the extra food generated without the FBN. However, we did not include that part in the study, as the presence of the FBN would not affect the outcome of such impact. Therefore, this data would not be relevant for the comparative analysis.

### 4.3. Recommendations

If one cannot measure the environmental impacts of food waste, one cannot manage them. In line with the results of previous studies, a more aligned approach on the evaluation criteria and the associated indicators would give more insight in which actions are more beneficial to address food waste [42]. This would facilitate the evaluation of food waste measures and predict what measures are more effective. New technologies, such as the application of Internet of Things (IoT)-based monitoring systems, could help us to more efficiently perform measurements along the food chain [47]. More complete information on the impacts of measures would make incentives for reducing food waste at various levels along the food chain more visible.

On the other hand, as regards data availability, it would be interesting to count on the public for quantitative and qualitative data on waste management systems at a regional level, with a view to conduct future studies in the field of environmental impact assessment and the reduction of food waste. This would increase the transparency of the recycling policies and would be useful to raise awareness on the environmental impact of the different waste management systems.

Regarding policy, the creation of new regulations—where possible—that encourage producers and distributors to recycle the food suitable for consumption at risk of not reaching the consumer would be highly recommended. This study has outlined the important hydrological benefits that this “food recycling” bring. We also have to keep in mind the social welfare that those political measures would mean to groups at risk of social exclusion and poverty. This is currently more and more important. The data published by the European Food Bank Federation warns that the demand for food has increased up to 50% compared to the pre-COVID-19 period, causing an increase in the activities of European food banks [48].

The present article demonstrates the positive environmental and social effects of the food banks and their redistribution and consumer education activities. Reinforcing this type of foundation would contribute to the creation of fairer and more environmentally friendly food systems, in line with the European Green Deal and its landmark Farm to Fork Strategy [2] and contributing to the fulfilment of the United Nations Sustainable Development Goals 2—Zero Hunger, 6—Clean Water and Sanitation and 12—Responsible Consumption and Production [49].

## 5. Conclusions

The comparative analysis carried out shows that the water use avoided by the rescue and utilization of food by the Food Bank of Navarra that would otherwise be wasted, in comparison to the water use associated to the activities of the FBN in the year 2018, is 1700 times higher. As a result, the activity of the FBN not only prevented the waste of 2.7 thousand tonnes of food fit for consumption, but also avoided the unnecessary waste of more than 3.2 million m^3^ of fresh water, the equivalent of filling 974 Olympic swimming pools. These water savings are mainly related to food production. These results highlight the importance, not only social but also environmental, of the food banks, since they prevent not only a large amount of greenhouse gases from being emitted into the atmosphere but also a large amount of water from being consumed and polluted. The more food is rescued and the greener the energy used in the facilities (wind, solar, except biofuels and hydropower) by the food banks, the greater the water savings will be. Finally, this paper calls for further research to objectively quantify the water-related and other impacts of food waste-reducing interventions along the entire chain using harmonized evaluation criteria and indicators.

## Figures and Tables

**Table 1:** Green, blue and grey water footprints (WF) of the Food Bank of Navarra in the year 2018 (m^3^/year).

Source of Consumption	Green WF (m^3^/year)	Blue WF (m^3^/year)	Grey WF (m^3^/year)	Total WF (m^3^/year)
**Operational water footprint**				
Facilities of the FBN Total operational water footprint	0 0	0 0	0 0	0 0
**Supply chain water footprint**				
Energy	0	134	0	134
Foodstuff transport	0	67	0	67
Personnel transport	0	40	0	40
Consumption of goods and services Total supply chain water footprint	1685 1685	0 241	0 0	1685 1926

## Appendix A

**Table A1:** Conversion factors for the different energy sources used by the energy providers to the Food Bank of Navarra: EMASP s. coop and Iberdrola Clientes in the year 2018.

Energy Source	Conversion Factor (Blue WF m^3^/Energy Unit)	Unit	Source
Solar	3090	GWh	[28]
Mini-hydroelectric	10,000	GWh	[28] ^1^
Diesel	0.03	GJ	[30]
Petrol	0.08	GJ	[30]
Cogeneration	684	GWh	[28]
Gas	580	GWh	[50] ^2,3^
Carbon	1552	GWh	[28]
Wind power	1.7	GWh	[51] ^3^
Hydroelectric	40,814	GWh	[28]
Nuclear	1569	GWh	[28]

^1^ Mini-hydroelectric plants have been considered to be a normal hydroelectric plant, with minimum levels of water consumption. ^2^ Natural gas combined cycles. ^3^ Average data for Spain, except for wind energy and combined cycle gas, whose data are global averages.

**Table A2:** Conversion factors for the different goods used by the Food Bank of Navarra in the year 2018.

Source of Consumption	Conversion Factor (Green WF m^3^/Unit)	Unit	Source
Wood	0.2	Kg of sawn lumber	[35] ^1^
Cardboard	171.9	Ton of cardboard	[35] ^1^

^1^ Joep F. Schyns has provided the specific factors for Spain from his study published in 2017 [35].

**Table A3:** Water use for the different waste management systems considered.

Energy Source	Source	Considered Water Use
Landfill	[52] ^1^	58.5 m^3^ of grey water per ton of dumping in landfills
Paper and cardboard	[53]	24 m^3^ of blue water per ton of recycled paper
Composting	MCP, Arazuri ^2^	0 m^3^ considering closed composting
Biomethanization	E-cogeneración cabanillas and HTN Biogas ^3^	0 m^3^
Light packaging	Sernaplas	0 m^3^
Glass	FCC ámbito	0 m^3^
Reject	-	-
Other	-	-

^1^ For landfill’s influence on the grey water footprint, U.S. standards of water quality have been considered. ^2^ Information provided by the Arazuri composting plant of the Pamplona Region Commonwealth (oral communication with Sandra Blazquez, May 2021). ^3^ Information provided by HTN Biogas (oral communication with Rubén Rodríguez, May 2021).

**Table A4:** Food redistributed by the Food Bank of Navarra in the year 2018 and bibliographic sources consulted for the water footprint estimation.

Food Category	Source Consulted for the Water Footprint Estimation
Fruits	[41]
Vegetables and legumes	[41]
Canned vegetables and legumes	[41]
Dairy products	[40]
Unclassified food	Weighted average
Beverages	[40,41,54]
Prepared dishes	[40,41,55]
Pastries, cookies and sweets	[40,41]
Condiments and sauces	[40,41]
Cereals, flour, bread and pasta	[41]
Meats	[40]
Snacks	[41]
Canned fruits	[41]
Eggs	[40]
Cocoa and chocolates	[41]
Coffees and infusions	[41]
Fish	[41]
Nuts	[41]
Children’s food	[41] and weighted average
